# Impact of obesity on early in-hospital postoperative outcomes following total knee arthroplasty in older adults: a comparative study of class I and class II obesity

**DOI:** 10.1007/s00402-025-05763-6

**Published:** 2025-04-24

**Authors:** David Maman, Merav Ben Natan, Yaron Berkovich

**Affiliations:** 1 Carmel Medical Center, Haifa, Israel; 2 Hillel Yaffe Academic School of Nursing, Hadera, Israel; 3 Technion - Israel Institute of Technology, Haifa, Israel

**Keywords:** Total knee arthroplasty, Obesity, Class I obesity, Class II obesity, In-hospital, Postoperative outcomes

## Abstract

**Introduction:**

The rising prevalence of obesity has increased the demand for total knee arthroplasty (TKA), but the impact on outcomes, particularly in Class I and II obesity, remains inconclusive. This study aimed to compare early in-hospital post-TKA outcomes in older patients with Class I (BMI 30–34.9 kg/m²) and Class II obesity (BMI 35–39.9 kg/m²).

**Materials and methods:**

Using data from the Nationwide Inpatient Sample (2016–2019), patients aged 65 and above who underwent TKA were categorized into Class I (*n* = 133,425) and Class II obesity groups (*n* = 122,432). Propensity score matching balanced baseline characteristics. Primary outcomes included in-hospital mortality and early postoperative complications.

**Results:**

Post-matching, a higher prevalence of type 2 diabetes was found in the Class II obesity group (32.5% vs. 29.5%, *P* = 0.001). The Class II group had a significantly higher risk of in-hospital mortality (9.004-fold, 95% CI: 3.57–22.68, *P* < 0.001), acute kidney injury (45.8% increase, 95% CI: 1.383–1.537, *P* < 0.001), and postoperative pneumonia (32.5% increase, 95% CI: 1.095–1.604, *P* = 0.004). The risk of blood loss anemia was 3.3% lower in the Class II group (95% CI: 0.946–0.988, *P* = 0.002). No significant differences were found in heart failure, acute coronary artery disease, pulmonary edema, venous thromboembolism, pulmonary embolism, and blood transfusion.

**Conclusions:**

More intensive monitoring and preventive measures may be necessary for patients with Class II obesity to mitigate the heightened risks associated with TKA compared to those with Class I obesity.

**Supplementary Information:**

The online version contains supplementary material available at 10.1007/s00402-025-05763-6.

## Introduction

Obesity, defined as having a body mass index (BMI) exceeding 30 kg/m², has become a widespread chronic condition globally, with its prevalence surging in recent years to epidemic levels. In the United States, one-third of adults are considered obese [1]. This trend poses a significant risk factor for osteoarthritis (OA), particularly affecting the knee joint [2], thereby increasing the demand for total knee arthroplasty (TKA) among obese individuals. A recent study has demonstrated that obese patients accounted for 23.2% of all TKAs performed [1].

The World Health Organization (WHO) categorizes obesity into three groups: Class I (moderate obesity) with a BMI of 30.0–34.9 kg/m², Class II (severe obesity) with a BMI of 35.0–39.9 kg/m², and Class III (morbid obesity) with a BMI of 40.0 kg/m² or higher [3]. While some studies indicate that obese and morbidly obese patients achieve post-TKA functional outcomes similar to their non-obese counterparts [4–7], others suggest poorer outcomes among morbidly obese individuals [8, 9].

In addition to functional outcomes, research suggests that obesity, especially morbid obesity, generally correlates with higher complication rates post-TKA [1, 5, 6, 8, 10–12]. However, some studies challenge this notion [11, 13–16]. Furthermore, there is inconsistency regarding the impact of obesity on mortality risk post-TKA [1, 13, 17].

Notably, there exists a paucity of research concerning post-TKA outcomes among patients with Class I (BMI 30–34.9 kg/m²) and Class II obesity (BMI 35–39.9 kg/m²). Gaining insights into the distinct challenges and risks faced by these subgroups is imperative for optimizing perioperative care and enhancing patient outcomes.

As the global population ages, individuals over 65 are projected to make up a significant portion of society by 2030, especially in Europe and Northern America [18]. Alongside this demographic shift, the prevalence of obesity among older adults is expected to rise [19]. Given that OA is common in older age groups, there is a pressing need to examine the outcomes of TKA in this demographic, which often has a higher burden of comorbidities. Therefore, this study will focus on older adults with Class I and Class II obesity to gain a better understanding of the complexities involved in managing TKA in this population. Specifically, it aims to compare early in-hospital post-TKA outcomes between these two groups of patients to provide insights into their unique challenges and needs.

## Methods

### Data source and study population

This study utilized data extracted from the Nationwide Inpatient Sample (NIS) dataset, the largest publicly available all-payer inpatient care database in the United States. The dataset, spanning the years 2016 to 2019, includes approximately 20% of inpatient stays from hospitals associated with the Healthcare Cost and Utilization Project (HCUP), capturing around seven million unweighted enrollments annually.

The study population comprised individuals aged 65 years and above who underwent elective primary TKA and had a BMI of 30 kg/m² or higher. We excluded patients with non-elective admissions or those who underwent surgery before admission to maintain data homogeneity. Patients who underwent TKA were identified using relevant ICD-10 procedure codes related to total knee replacement. The comprehensive list of included codes is provided in the appendix.

Patients were stratified into two groups based on their BMI at the time of admission. Group 1 comprised patients with a BMI ranging from 30 to 34.9 (Class I obesity), while Group 2 consisted of patients with a BMI ranging from 35 to 39.9 (Class II obesity). The total number of patients included in the study was 255,857.

Patients with Class III obesity (BMI ≥ 40 kg/m²) were not included in our study as a comparator group. This decision was based on the limited representation of such patients in our dataset, with only a few hundred cases identified. This low prevalence is likely attributable to restricted access to TKA for patients with severe obesity. Including this small subset would have precluded statistically meaningful analyses or robust conclusions. Furthermore, the inclusion of such a limited sample could introduce bias and compromise the reliability of our findings. Therefore, we focused on patients with Class I and Class II obesity, which were better represented in the dataset and allowed for more robust and reliable analyses.

### Propensity score matching

To minimize confounding factors, propensity score matching was performed using MATLAB. We matched patients with a BMI of 30 to 34.9 to those with a BMI of 35 to 39.9 based on several characteristics, including hospital size, patient location (urban/rural), median household income, hospital region, total discharges within the NIS dataset, comorbidities, payer type, sex, and race. This process resulted in a balanced dataset ensuring that any observed differences in outcomes could be attributed to the BMI category rather than underlying patient demographics or comorbidities.

### Outcome measures

The primary outcome measures included in-hospital mortality and in-hospital post-TKA complications.

### Data analyses

Descriptive statistics were employed to summarize patient demographics and baseline characteristics. Statistical analyses were performed using SPSS Statistics version 26 (IBM Corp., Armonk, NY, USA), which included chi-square tests, independent samples t-tests, and risk ratio calculations. To account for potential residual differences following propensity score matching, a multivariate logistic regression analysis was conducted to evaluate the impact of comorbidities on primary outcomes and complications. A p-value of less than 0.05 was considered statistically significant.

### Ethical considerations

This study received exempt status from the institutional review board due to the de-identified nature of the NIS dataset. The requirement for informed consent was waived.

## Results

The analysis revealed significant differences between Class I (BMI 30–34.9 kg/m ^2^) and Class II (BMI 35–39.9 kg/m ^2^) obesity groups. Table 1 presents a comparative examination of sociodemographic characteristics. Notably, a higher total number of TKA surgeries were observed among patients in the Class I obesity group compared to those in the Class II obesity group. The average age of patients in the Class I obesity group was slightly higher, compared to the Class II obesity group (72.21 vs. 71.14 years, *P* < 0.001). The Class II obesity group displayed a higher percentage of female patients compared to the Class I obesity group, with proportions of 65.9% and 60.5%, respectively (*P* < 0.001).

Furthermore, Medicare emerged as the primary payer for TKA procedures in both the Class I obesity group and the Class II obesity group, with a slightly higher proportion in the Class I obesity group. The racial distribution showed that the majority in both BMI groups were White, with slight disparities observed between the two groups. Notably, the Class II obesity group had a higher proportion of Black patients compared to the Class I obesity group. Additionally, variations in income distribution were evident across the BMI categories, with slight differences noted across several income brackets.

**Table 1:** Comparison of sociodemographic characteristics: class I vs. class II obesity TKA patients

Characteristic	Class I obesity *n* = 133,425	Class II obesity *n* = 122,432	*P*-value
Age (mean, years)	72.2	71.1	< 0.001
Female (%)	60.5	65.9	< 0.001
Payer - Medicare (%**)**	84.9	84	< 0.001
Payer - Medicaid (%**)**	0.8	0.8	
Payer - Private (%**)**	12.4	13.9	
Payer - Other (including self-pay) (%)	1.9	1.3	
Race - White (%)	82.8	82.9	< 0.001
Race - Black (%)	7.4	8.4	
Race - Hispanic (%)	6.1	5.5	
Race - Asian or Pacific Islander (%)	1.2	0.8	
Race - Native American (%)	0.3	0.3	
Race - Other (%)	2.1	2	
Median Household Income: 0-25th Percentile (%)	19.7	20.5	< 0.001
Median Household Income 26th-50th Percentile (%)	25.7	27.1	
Median Household Income 51th-75th Percentile (%)	28.4	28.5	
Median Household Income 76th-100th Percentile (%)	26.1	24	

The analysis of comorbidities among patients revealed significant differences between the two BMI groups, with several notable variations evident, as detailed in Table 2. Among these differences, the most substantial were observed in the prevalence of type 2 diabetes, with a markedly higher prevalence among patients in the Class II (32.5%) compared to those in the Class I obesity group (26.9%). Similarly, chronic lung disease exhibited a notable disparity, with patients in the Class II demonstrating a higher prevalence (7.8%) than those in the Class I obesity group (6.7%). Additionally, patients in the Class II showed a higher prevalence of hypertension (67.9%) compared to those in the Class I obesity group (65%), while patients in the Class I exhibited a higher prevalence of dyslipidemia (62.1%) relative to the Class II obesity group (60.3%). Furthermore, differences in the prevalence of other comorbidities, including chronic anemia, alcohol abuse, mental disorders, Parkinson’s disease, osteoporosis, renal disease, and congestive heart failure (CHF), were observed between the two BMI groups.

**Table 2:** Prevalence of comorbidities: class I vs. class II obesity TKA patients

	Class I obesity *n* = 133,425	Class II obesity *n* = 122,432	*P*-value
Hypertension Diagnosis (%)	65	67.9	< 0.001
Dyslipidemia Diagnosis (%)	62.1	60.3	< 0.001
Chronic Anemia (%)	6.6	6.2	< 0.001
Alcohol Abuse (%)	0.8	0.7	< 0.01
Mental Disorders (%)	27.4	28.6	< 0.001
Parkinson Disease (%)	0.6	0.5	< 0.05
Osteoporosis Diagnosis (%)	5.1	4	< 0.001
Type 2 Diabetes (%)	26.9	32.5	< 0.001
Renal Disease (%)	11.8	12.6	< 0.001
Congestive Heart Failure (%)	1.5	2	< 0.001
Chronic Lung Disease (%)	6.7	7.8	< 0.001

After propensity score matching, the two BMI groups were statistically comparable across all parameters in Table 3, with the exception of type 2 diabetes. This method effectively balanced baseline characteristics between the Class I and Class II obesity groups. However, a slight but significant difference in the prevalence of type 2 diabetes persisted, with 32.5% in Class II and 29.5% in Class I. While this difference is modest, it underscores the strong association between obesity and type 2 diabetes, which may not have been entirely accounted for by the propensity score matching.

To further address this concern, we performed a multivariate logistic regression analysis to assess the potential influence of residual differences in comorbidities, including type 2 diabetes, on outcomes and complications. This analysis included type 2 diabetes and other key comorbidities as independent variables, with primary outcomes such as blood transfusion, acute kidney injury, and in-hospital mortality analyzed as dependent variables. The results showed that type 2 diabetes was not a significant predictor of any primary outcomes or complications. These findings suggest that the propensity score matching effectively minimized confounding factors and that the slight difference in type 2 diabetes prevalence did not substantially affect the study results.

**Table 3:** Patient characteristics after propensity score matching

	Class I obesity *n* = 122,060	Class II obesity *n* = 122,060	*P*-value
Age (mean, years)	71.16	71.1	0.1
Female (%)	65.6	65.8	0.1
Hypertension Diagnosis %	67.6	67.9	0.06
Dyslipidemia Diagnosis (%)	60.3	60.3	0.5
Chronic Anemia (%)	6.3	6.2	0.1
Alcohol Abuse (%)	0.8	0.7	0.08
Mental Disorders (%)	28.4	28.6	0.32
Parkinson Disease (%)	0.6	0.5	0.22
Type 2 Diabetes (%)	29.5	32.5	0.001
Renal Disease (%)	12.4	12.6	0.09
Congestive Heart Failure (%)	1.9	2	0.36
Chronic Lung Disease (%)	7.8	7.8	0.44

**Fig. 1:**
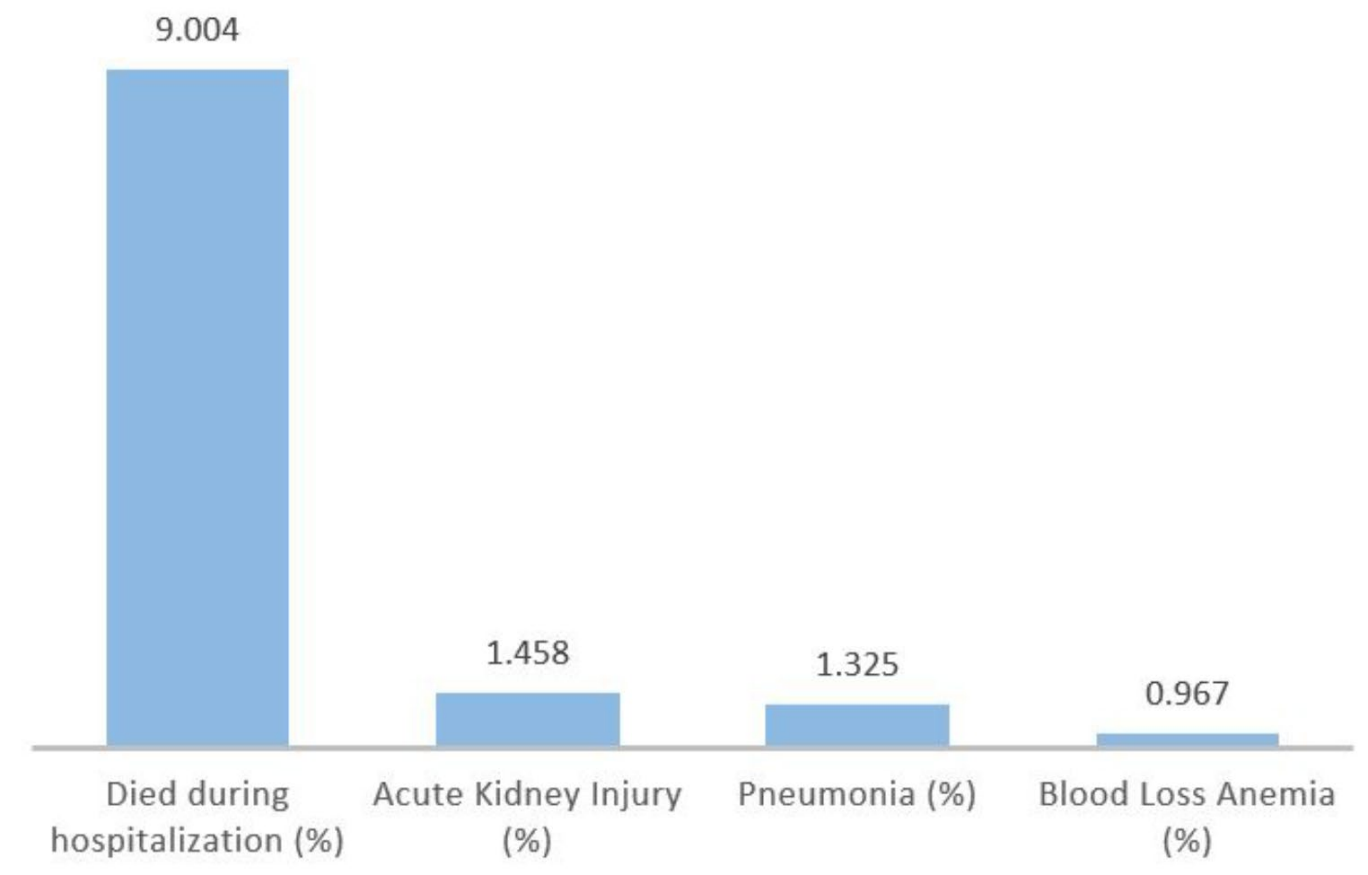
Increased risk of early in-hospital postoperative complications in class ii obesity group compared to class i obesity group in propensity score-matched cohorts

In our examination of early in-hospital postoperative complications among propensity score-matched cohorts, we gained noteworthy insights when comparing patients from the Class I and Class II obesity groups. As shown in Table 4, our analysis revealed no statistically significant differences in the incidence of heart failure, acute coronary artery disease, pulmonary edema, venous thromboembolism, pulmonary embolism, and blood transfusion between the two BMI groups. However, when we extended our analysis to include additional complications, as detailed in Fig. 1, distinctions between the groups emerged.

**Table 4:** Comparison of postoperative complications between obese class I and obese class II in Propensity score-matched cohorts

	Class I obesity *n* = 122,060	Class II obesity *n* = 122,060	*P*-value
Heart Failure (%)	0.20	0.20	0.28
Acute Coronary Artery Disease (%)	0.10	0.10	0.81
Pulmonary Edema (%)	0.10	0.10	0.56
Venous Thromboembolism (%)	0.37	0.40	0.08
Pulmonary Embolism (%)	0.24	0.25	0.42
Blood Transfusion (%)	1.17	1.22	0.25

As shown in Fig. 1, the risk estimates highlight the increased likelihood of certain early in-hospital postoperative complications when comparing patients in the Class I and Class II obesity groups. The risk of in-hospital mortality was significantly higher in the Class II obesity group, with a 9.004-fold increase compared to the Class I obesity group (95% CI: 3.57–22.68, *P* < 0.001). Additionally, the risk of developing acute kidney injury was 45.8% higher in the Class II obesity group relative to the Class I obesity group (Risk Estimate: 1.458, 95% CI: 1.383–1.537, *P* < 0.001). The risk of postoperative pneumonia was 32.5% higher in the Class II obesity group compared to the Class I obesity group (Risk Estimate: 1.325, 95% CI: 1.095–1.604, *P* = 0.004). Conversely, the risk of developing blood loss anemia was 3.3% lower in the Class II obesity group compared to the Class I obesity group (Risk Estimate: 0.967, 95% CI: 0.946–0.988, *P* = 0.002).

## Discussion

This study compares early in-hospital post-TKA outcomes in older patients with Class I (BMI 30–34.9 kg/m²) and Class II obesity (BMI 35–39.9 kg/m²). We identified several differences, with Class I showing more surgeries, suggesting a larger pool seeking intervention for knee osteoarthritis. Class II patients were younger, with a higher female percentage, aligning with existing literature [11, 14, 16]. Obesity’s association with knee osteoarthritis leads to earlier surgery, especially in women who have a higher prevalence of both conditions. Additionally, more Black patients in Class II suggest potential healthcare access disparities. Furthermore, income variations across BMI categories, possibly reflect the impact of socioeconomic factors on orthopedic surgery utilization.

In terms of comorbidities, substantial disparities were observed between the two BMI groups. The Class II obesity group demonstrated a significantly higher prevalence of type 2 diabetes, hypertension, and chronic lung disease compared to the Class I obesity group. These findings echo previous research highlighting the strong association between severe obesity and cardiometabolic disorders [20].

Using propensity score matching, we balanced baseline characteristics between the Class I and Class II obesity groups, except for type 2 diabetes, indicating effective control of confounding variables. However, a slight but significant difference in the prevalence of type 2 diabetes persisted. This modest disparity underscores the complex relationship between obesity and metabolic health. The higher prevalence of type 2 diabetes in the Class II obesity group likely reflects the profound metabolic dysregulation characteristic of severe obesity, which may predispose patients to poorer post-TKA outcomes [21]. Despite this, our multivariate logistic regression analysis indicated that type 2 diabetes was not a significant predictor of any primary outcomes or complications, suggesting that the residual difference in diabetes prevalence did not substantially affect the study results.

Our post-matching examination indicated a heightened likelihood of specific complications in the Class II obesity group, notably, a significantly increased risk of in-hospital mortality. This aligns with studies such as Mohamed et al. [1] and D’Apuzzo et al. [13], which have found associations between obesity and mortality, primarily focusing on morbid obesity. Our findings suggest that the risks of mortality may extend to less severe obesity as well.

Moreover, patients classified under Class II obesity demonstrated higher risk of acute kidney injury compared to their Class I counterparts. This observation aligns with Ward et al.‘s [12] research, which emphasized a heightened likelihood of complications, including acute kidney injury, associated with higher BMI, particularly beyond a BMI of 40, despite a relatively minor absolute risk difference. Furthermore, we found that patients in the Class II obesity group faced a substantially higher risk of postoperative pneumonia than those in the Class I group. Similarly, D’Apuzzo et al. [13] highlighted an independent association between morbid obesity and a heightened risk of select in-hospital postoperative complications. Our findings suggest that these risks extend to individuals with less severe obesity as well. This contradicts studies such as Belmont et al. [22], which suggested that only patients with morbid obesity had a significantly increased risk of developing any complication.

On the contrary, our study revealed a lower incidence of blood loss anemia in the Class II obesity group compared to Class I obesity counterparts. This finding resonates with previous research indicating reduced transfusion rates among obese and morbidly obese patients undergoing TKA [14, 16]. Similarly, Tió et al. [15] observed comparable blood loss and transfusion requirements between severely and morbidly obese individuals and non-obese or moderately obese patients undergoing TKA. This trend suggests that meticulous surgical techniques and enhanced hemostatic measures are more diligently employed in obese patients, potentially mitigating the need for transfusions. However, it is important to note that, according to the NIS database coding conventions, if a patient receives a blood transfusion during or because of surgery, they are automatically assigned a diagnosis of blood loss anemia. This ensures that cases requiring transfusion are accurately captured under the blood loss anemia category. As such, the lower incidence of blood loss anemia observed in the Class II obesity group reflects the true recorded rates within the dataset and is not due to missed intra-operative or post-operative transfusions. This coding practice aligns with standardized hospital reporting protocols and further supports the robustness of our findings.

We found no significant differences in heart failure, acute coronary artery disease, pulmonary edema, venous thromboembolism, pulmonary embolism, or blood transfusion between Class I and Class II obesity groups. This aligns with prior research indicating similar thromboembolic risks among obese and non-obese TKA patients [11, 14, 16]. Comparable venous thromboembolism rates suggest effective prophylactic measures in our cohort. Wallace et al. [17] also noted no obesity association with acute myocardial infarction. Tió et al. [15] further reported no elevated blood loss or transfusion needs in severely and morbidly obese TKA patients.

Our study has several limitations. Despite using propensity score-matched analysis to address selection bias, it may still be susceptible to residual confounding factors inherent in observational studies. Additionally, it lacks the capacity to establish causality between obesity and post-TKA outcomes. Inherent biases in retrospective analyses, such as incomplete medical records, may impact the validity of our findings. Furthermore, this study covers only the in-hospital postoperative period, which is a very narrow timeframe. The outcomes described as “early” are limited to in-hospital results and do not include complications that may develop post-discharge, such as those within 30 days. This limitation should be addressed, as it is possible that the Class I obesity group experienced similar complications later in the postoperative period. Another limitation is the exclusion of a comparison with non-obese patients, thus limiting the broader applicability of our findings. However, this comparison was not included due to the specific focus of our research on understanding the nuances between different classes of obesity.

## Conclusions

Our findings indicate that older TKA patients with Class II obesity face significantly higher risks of in-hospital mortality, acute kidney injury, and postoperative pneumonia compared to those with Class I obesity. These results underscore the critical role of obesity severity in predicting adverse outcomes following TKA, suggesting that more nuanced distinctions within obesity categories should be considered.

The heightened risks associated with Class II obesity highlight the need for personalized perioperative care strategies to mitigate adverse outcomes in this subgroup of patients. Healthcare providers should adopt approaches that account for these distinctions within obesity classifications when planning and managing TKA procedures. Furthermore, addressing socioeconomic factors and healthcare disparities is crucial to improving outcomes across all obesity categories in orthopedic surgery.

Future research should focus on validating these findings through prospective studies, exploring the specific mechanisms underlying outcomes disparities among different obesity classes, and incorporating comparisons with non-obese populations to enhance the applicability of clinical strategies in managing TKA outcomes.

## Electronic Supplementary Material

Below is the link to the electronic supplementary material.

Supplementary Material 1

## Data Availability

The data that support the findings of this study are available from the corresponding author upon reasonable request.

## References

[1] Mohamed NS, Wilkie WA, Remily EA, Dávila Castrodad IM, Jean-Pierre M, Jean-Pierre N, Gbadamosi WA, Halik AK, Delanois RE (2022) The rise of obesity among total knee arthroplasty patients. J Knee Surg 35. 10.1055/s-0040-1710566 10.1055/s-0040-1710566 32443160

[2] Singer SP, Dammerer D, Krismer M, Liebensteiner MC (2018) Maximum lifetime body mass index is the appropriate predictor of knee and hip osteoarthritis. Arch Orthop Trauma Surg 138:99–103. 10.1007/s00402-017-2825-5 29079909 10.1007/s00402-017-2825-5 PMC5754409

[3] Sue R, Jitnarin N, Vidoni ML, Kaipust CM, Brown AL (2019) Epidemiology of adult obesity. Lifestyle medicine, 3rd edn. CRC, pp 455–471

[4] Baghbani-Naghadehi F, Armijo-Olivo S, Prado CM, Gramlich L, Woodhouse LJ (2022) Does obesity affect patient-reported outcomes following total knee arthroplasty? BMC Musculoskelet Disord 23:55–58. 10.1186/s12891-022-04997-4 35039019 10.1186/s12891-022-04997-4 PMC8764810

[5] Boyce L, Prasad A, Barrett M, Dawson-Bowling S, Millington S, Hanna SA, Achan P (2019) The outcomes of total knee arthroplasty in morbidly obese patients: a systematic review of the literature. Arch Orthop Trauma Surg 139:553–560. 10.1007/s00402-019-03127-5 30778723 10.1007/s00402-019-03127-5 PMC6420900

[6] Chen JY, Lo NN, Chong HC et al (2016) The influence of body mass index on functional outcome and quality of life after total knee arthroplasty. Bone Joint J 98–B:780–785. 10.1302/0301-620X.98B6.35709 27235520 10.1302/0301-620X.98B6.35709

[7] Singh V, Yeroushalmi D, Lygrisse KA, Simcox T, Long WJ, Schwarzkopf R (2022) The influence of obesity on achievement of a ‘forgotten joint’ following total knee arthroplasty. Arch Orthop Trauma Surg 142:491–499. 10.1007/s00402-021-03840-0 33661386 10.1007/s00402-021-03840-0

[8] Hakim J, Volpin G, Amashah M, Alkeesh F, Khamaisy S, Cohen M, Ownallah J (2020) Long-term outcome of total knee arthroplasty in patients with morbid obesity. Int Orthop 44:95–104. 10.1007/s00264-019-04378-y 31372812 10.1007/s00264-019-04378-y

[9] Maniar RN, Maniar PR, Singhi T, Gangaraju BK (2018) WHO class of obesity influences functional recovery post-TKA. Clin Orthop Surg 10:26–29. 10.4055/cios.2018.10.1.26 29564044 10.4055/cios.2018.10.1.26 PMC5851851

[10] Husted H, Jørgensen CC, Gromov K, Kehlet H, Lundbeck, Foundation Center for Fast-track Hip and Knee Replacement Collaborative Group (2016) Does BMI influence hospital stay and morbidity after fast-track hip and knee arthroplasty? Acta Orthop 87:466–472. 10.1080/17453674.2016.1203477 27347785 10.1080/17453674.2016.1203477 PMC5016904

[11] Abdel MP, Ast MP, Lee YY, Lyman S, Della Valle AG (2014) All-cause in-hospital complications and urinary tract infections increased in obese patients undergoing total knee arthroplasty. J Arthroplasty 29:1430–1434. 10.1016/j.arth.2014.02.013 24703783 10.1016/j.arth.2014.02.013

[12] Ward DT, Metz LN, Horst PK, Kim HT, Kuo AC (2015) Complications of morbid obesity in total joint arthroplasty: risk stratification based on BMI. J Arthroplasty 30:42–46. 10.1016/j.arth.2015.03.045 26117070 10.1016/j.arth.2015.03.045

[13] D’Apuzzo MR, Novicoff WM, Browne JA (2015) The John Insall Award: morbid obesity independently impacts complications, mortality, and resource use after TKA. Clin Orthop Relat Res 473:57–63. 10.1007/s11999-014-3668-9 24818736 10.1007/s11999-014-3668-9 PMC4390915

[14] Gurunathan U, Pym A, Anderson C, Marshall A, Whitehouse SL, Crawford RW (2018) Higher body mass index is not a risk factor for in-hospital adverse outcomes following total knee arthroplasty. J Orthop Surg 26. 10.1177/2309499018802429 10.1177/2309499018802429 30270748

[15] Tió M, Basora M, Rios J, Sánchez-Etayo G, Bergé R, Sastre S, Lozano L (2018) Severe and morbid obesity and transfusional risk in total knee arthroplasty: an. Observational Study Knee 25:923–931. 10.1016/j.knee.2018.07.005 30029995 10.1016/j.knee.2018.07.005

[16] Woon CY, Piponov H, Schwartz BE, Moretti VM, Schraut NB, Shah RR, Goldstein WM (2016) Total knee arthroplasty in obesity: in-hospital outcomes and national trends. J Arthroplasty 31:2408–2414. 10.1016/j.arth.2016.04.028 27259393 10.1016/j.arth.2016.04.028

[17] Wallace G, Judge A, Prieto-Alhambra D, de Vries F, Arden NK, Cooper C (2014) The effect of body mass index on the risk of post-operative complications during the 6 months following total hip replacement or total knee replacement surgery. Osteoarthritis Cartilage 22:918–927. 10.1016/j.joca.2014.04.013 24836211 10.1016/j.joca.2014.04.013

[18] Wilmoth JR, Bas D, Mukherjee S, Hanif N (2023) World social report 2023: leaving no one behind in an ageing world. UN

[19] Kalish VB (2016) Obesity in older adults. Prim Care 43:137–144. 10.1016/j.pop.2015.10.002 26896206 10.1016/j.pop.2015.10.002

[20] Valenzuela PL, Carrera-Bastos P, Castillo-García A, Lieberman DE, Santos-Lozano A, Lucia A (2023) Obesity and the risk of cardiometabolic diseases. Nat Rev Cardiol 20:475–494. 10.1038/s41569-023-00847-5 36927772 10.1038/s41569-023-00847-5

[21] Ruze R, Liu T, Zou X, Song J, Chen Y, Xu R, Xu Q (2023) Obesity and type 2 diabetes mellitus: connections in epidemiology, pathogenesis, and treatments. Front Endocrinol (Lausanne) 14. 10.3389/fendo.2023.1161521 10.3389/fendo.2023.1161521 PMC10161731 37152942

[22] Belmont PJ Jr, Goodman GP, Waterman BR, Bader JO, Schoenfeld AJ (2014) Thirty-day postoperative complications and mortality following total knee arthroplasty: incidence and risk factors among a national sample of 15,321 patients. JBJS 96:20–26. 10.2106/JBJS.M.00018 10.2106/JBJS.M.00018 24382720

