# Supplementary material for: Impact of obesity on early in-hospital postoperative outcomes following total knee arthroplasty in older adults: a comparative study of class I and class II obesity
Source: Arch Orthop Trauma Surg. 2025 Apr 24;145(1):262. doi: 10.1007/s00402-025-05763-6 (PMC12021722; doi:10.1007/s00402-025-05763-6)
Supplement: Supplementary file 1 — Supplementary Material 1 [file 402_2025_5763_MOESM1_ESM.docx]

| ICD 10 CODES / PROCEDURE CODE |  |
| --- | --- |
| I5021, I5031, I5033, I5041, I5043 | Heart Failure |
| N170, N171, N172, N178, N179 | Acute Kidney Injury |
| I2101, I2102, I2109, I211, I2119, I2111, I212, I2129, I213, I214, I219 | Acute Coronary Artery Disease |
| I60, I61, I62, I63, I650, I688, O873, O2250, O2251, O2252 | Stroke |
| J810, J811, I501 | Pulmonary Edema |
| I10(start with) | Hypertension |
| D62 (start with) | Blood Loss Anemia |
| J189, J159, J22 | Pneumonia |
| I2602, I2609, I2692, I2699 | Pulmonary Embolism |
| I82401, I82402, I82403, I82409, I82411, I82412, I82413, I82419, I82421, I82422, I82423, I82429 | DVT |
| E78(start with) | Dyslipidemia |
| G473 | Obstructive Sleep Apnea |
| D64(start with) | Chronic Anemia |
| F10 | Alcohol Abuse History |
| M81, M82 | Osteoporosis |
| F (start with) | Mental Disorders |
| G20 (start with) | Parkinson Disease |
| E11 (start with) | Type 2 Diabetes Mellitus |
| N18 (start with) | Chronic Kidney Disease |
| I500, I501, I509 | Congestive Heart Failure |
| J44 (start with) | Chronic Lung Disease |
|  |  |
